# DecodeME: community recruitment for a large genetics study of myalgic encephalomyelitis / chronic fatigue syndrome

**DOI:** 10.1186/s12883-022-02763-6

**Published:** 2022-07-19

**Authors:** Andy Devereux-Cooke, Sian Leary, Simon J. McGrath, Emma Northwood, Anna Redshaw, Charles Shepherd, Pippa Stacey, Claire Tripp, Jim Wilson, Margaret Mar, Danielle Boobyer, Sam Bromiley, Sonya Chowdhury, Claire Dransfield, Mohammed Almas, Øyvind Almelid, David Buchanan, Diana Garcia, John Ireland, Shona M. Kerr, Isabel Lewis, Ewan McDowall, Malgorzata Migdal, Phil Murray, David Perry, Chris P. Ponting, Veronique Vitart, Jareth C. Wolfe

**Affiliations:** 1 c/o DecodeME, MRC Human Genetics Unit, University of Edinburgh, Edinburgh, EH4 2XU UK; 2 Action for ME, 42 Temple Street, Keynsham, BS31 1EH UK; 3 MRC Human Genetics Unit, University of Edinburgh, Edinburgh, EH4 2XU UK

**Keywords:** Myalgic encephalomyelitis, Co-production, Genome-wide association study, Patient and Public Involvement

## Abstract

**Background:**

Myalgic encephalomyelitis / chronic fatigue syndrome (ME/CFS) is a common, long-term condition characterised by post-exertional malaise, often with fatigue that is not significantly relieved by rest. ME/CFS has no confirmed diagnostic test or effective treatment and we lack knowledge of its causes. Identification of genes and cellular processes whose disruption adds to ME/CFS risk is a necessary first step towards development of effective therapy.

**Methods:**

Here we describe DecodeME, an ongoing study co-produced by people with lived experience of ME/CFS and scientists. Together we designed the study and obtained funding and are now recruiting up to 25,000 people in the UK with a clinical diagnosis of ME/CFS. Those eligible for the study are at least 16 years old, pass international study criteria, and lack any alternative diagnoses that can result in chronic fatigue. These will include 5,000 people whose ME/CFS diagnosis was a consequence of SARS-CoV-2 infection. Questionnaires are completed online or on paper. Participants’ saliva DNA samples are acquired by post, which improves participation by more severely-affected individuals. Digital marketing and social media approaches resulted in 29,000 people with ME/CFS in the UK pre-registering their interest in participating. We will perform a genome-wide association study, comparing participants’ genotypes with those from UK Biobank as controls. This should generate hypotheses regarding the genes, mechanisms and cell types contributing to ME/CFS disease aetiology.

**Discussion:**

The DecodeME study has been reviewed and given a favourable opinion by the North West – Liverpool Central Research Ethics Committee (21/NW/0169). Relevant documents will be available online (www.decodeme.org.uk). Genetic data will be disseminated as associated variants and genomic intervals, and as summary statistics. Results will be reported on the DecodeME website and via open access publications.

## Background

Myalgic Encephalomyelitis/Chronic Fatigue Syndrome (ME/CFS) is a chronic disease affecting an estimated 250,000 people in the UK and approximately 1.5 million in the USA [1, 2]. The disease is characterised by substantial reduction or impairment of activity levels and is associated with high levels of disability and poor quality of life [3, 4]. ME/CFS symptoms include, but are not limited to, post-exertional malaise (PEM) and profound fatigue unassuaged by sleep [4, 5]. Approximately one-in-four people with ME/CFS are house-bound [6]. Despite its high cost to patients, their families and the economy, we know less about the causes of ME/CFS and how to treat it effectively than we do about many rarer and less disabling diseases [3, 4, 7]. ME/CFS is often diagnosed subsequent to infection (such as with Epstein Barr virus) [8]. A substantial minority of people infected with the COVID-19 coronavirus (SARS-CoV-2) may eventually be diagnosed with ME/CFS [9].

There is no biomolecular explanation why some people are at greater risk of being diagnosed with ME/CFS. This is despite many studies highlighting changes in biomolecules or cell types as being characteristic of ME/CFS [10]. For two reasons, such findings have yet to shed light on ME/CFS’s causal mechanisms. Firstly, only rarely are these studies independently replicated, which casts doubt on their explanatory power. Secondly, even when molecules or cell types distinguish people with ME/CFS from others, they could reflect secondary consequences of disease rather than its cause. Separating cause from effect in disease aids in the discovery of therapeutic interventions that improve disease outcomes.

Any significant genetic differences between people with—and those without—ME/CFS must reflect a biological cause of the illness. This is because DNA (unlike other biomolecules) remains unaffected by disease. Genetics is thus our preferred approach to identify molecules and cellular pathways that causally alter ME/CFS risk. Although many genes have been proposed to explain ME/CFS risk, none have survived independent replication using larger, and thus better powered, data sets [11]. This lack of replication could be explained by ME/CFS risk having no genetic contribution. However, this appears to be untrue: having a close (first- or second-degree) or distant (third-degree) relative with CFS significantly increases one’s own risk of the same diagnosis [12]. Nevertheless, ME/CFS diagnoses do not follow a predictable inheritance pattern, implying that ME/CFS is a complex multifactorial disorder whose risk is increased by many genetic variants.

In 2012, the CFS/ME Research Collaborative (CMRC) was formed whose aim was to catalyse a step-change in the amount and quality of ME/CFS research. By 2018 the CMRC, which now included a patient advisory group, decided to support an ME/CFS genome-wide association study (GWAS) because its results are unaffected by pre-existing biological assumptions or hypotheses, making it ideal for discovering genetic causes of disease and new biology [13]. By 2020 the DecodeME partnership of researchers, people with ME/CFS and carers was initiated and funding for the GWAS secured. The project’s aims are to predict genes, biological pathways and cell-types directly implicated in ME/CFS and to determine whether the genetics of ME/CFS overlaps with other diseases. In this way, we intend to generate strong scientific leads that researchers can pursue with future experiments. DecodeME hopes that this work ultimately will lead to the development of diagnostic tests and targeted treatments.

DecodeME’s three principal objectives are to: 1) Co-produce the world’s largest cohort of individuals clinically diagnosed with ME/CFS, to donate saliva samples for DNA extraction; 2) Undertake the world’s first well-powered (25,000 case) GWAS of ME/CFS; and, 3) Curate an open (managed) resource of genotype and phenotype data for these 25 k people with ME/CFS, consented to be invited to take part in future epidemiological, genetic, biomolecular and clinical trial and other clinical studies.

## Methods

DecodeME is a case–control GWAS, with collection of saliva samples from cases for DNA extraction and genetic analysis, and detailed phenotype data initially through questionnaires and, when granted consent, longitudinally through NHS electronic health records (EHR) data linkage. Questionnaires are delivered online using digital infrastructure. For people without internet access or who have difficulties using computers we provide an option for a paper-based version of the study documents delivered by post. For the GWAS an individual is automatically categorised as case (or non-case) from their questionnaire answers and Canadian Consensus or IOM/NAM inclusion/exclusion criteria [4, 14]. A new DecodeME questionnaire was developed from these published criteria, with iterative amendments resulting from active Patient and Public Involvement. The study began on September 1 2020 and its current funding ends on August 31 2024.

The partnership intends to recruit at least 20,000 ME/CFS cases whose initial symptoms arose prior to the COVID-19 pandemic. Recruitment of a further 5,000 ME/CFS diagnosed cases whose symptoms arose following SARS-CoV-2 infection was agreed by funders in 2021. Comparison of the two sub-cohorts will permit investigation of whether they share genetic risk factors. Cases whose initial ME/CFS symptoms arose following SARS-CoV-2 infection will not have been hospitalised as a result of SARS-CoV-2 infection and not informed of heart or lung damage as a result of COVID-19.

**Fig. 1:**
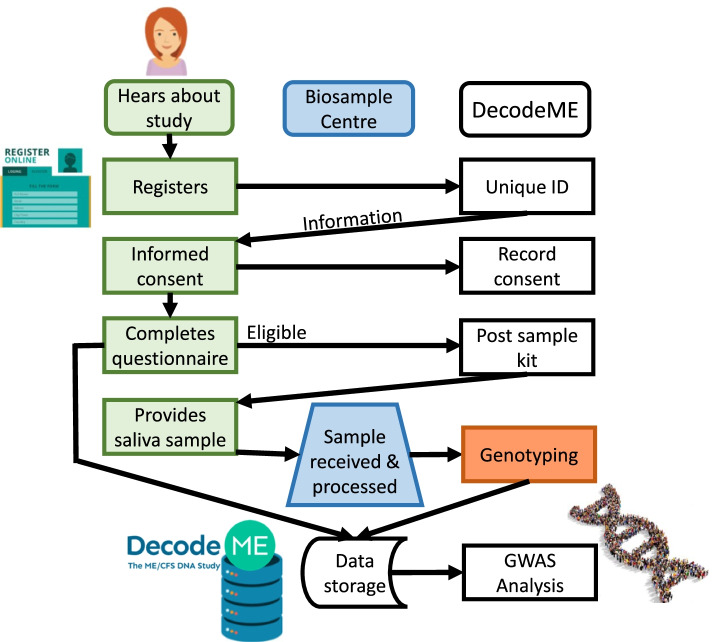
DecodeME study design involving the potential participant (green shapes), the DecodeME team (white), the NIHR Biosample Centre (blue) and the genotyping provider (brown), ThermoFisher/Affymetrix. Participants are told that they are not obliged to consent to participate in the study. At any point potential participants can contact DecodeME with questions. If progress is not made on consent or the questionnaire or returning the saliva collection kit then DecodeME contacts the participant. If DNA from the sample fails quality control then a second kit is sent to the participant

Participants’ saliva DNA samples are acquired via the postal system, a design intended to improve participation by more severely-affected individuals. The DecodeME study design is summarised in Fig. 1. A DNA sub-aliquot from each consenting participant is being stored for future whole genome sequencing. Control genotype data have been acquired from UK Biobank matched participants unaffected by ME/CFS [15]. Matching will occur based on sex-at-birth and genetic ancestry. Exclusion criteria (below) will be applied to control UK Biobank individuals. Control individuals will, additionally, neither have self-reported CFS symptoms nor answered “Yes” to the following question: “Have you ever had Chronic Fatigue Syndrome or Myalgic Encephalomyelitis (M.E.)?”.

### Inclusion criteria

Study participants will: i) be at least 16 years of age; ii) report a diagnosis of ME or CFS (or ME/CFS or CFS/ME) given by a health professional; iii) have given their informed consent; and iv) conform to case definition according to the Canadian Consensus and/or IOM/NAM Criteria. There is no upper age limit.

### Non-inclusion criteria

Exclusion criteria are: (i) the lack of capacity to provide informed consent, (ii) any alternative diagnoses including major psychiatric illness (e.g. bipolar disorder or schizophrenia) that can result in chronic fatigue, as explicit in the Canadian Consensus and IOM/NAM criteria[4, 14]. (iii) Importantly, ‘self-diagnosis’ is ruled out: participants will be required to report their diagnosis of ME or CFS by a health professional.

### Identifying participants

DecodeME recruited its first participants in January 2022 and will continue recruitment until August 2024, or until the target number is reached. Figure 1 illustrates how we manage participants’ enrolment, including their consent for participation in the DecodeME Study. Our overall *spit-and-post* study design drew inspiration both from the “snowball sampling” approach of *Genes for Good* that used social media to engage a diverse participant pool [16] and from the focused and intense social and traditional media campaign of the GLAD (Genetic Links to Anxiety and Depression) study [17].

**Fig. 2:**
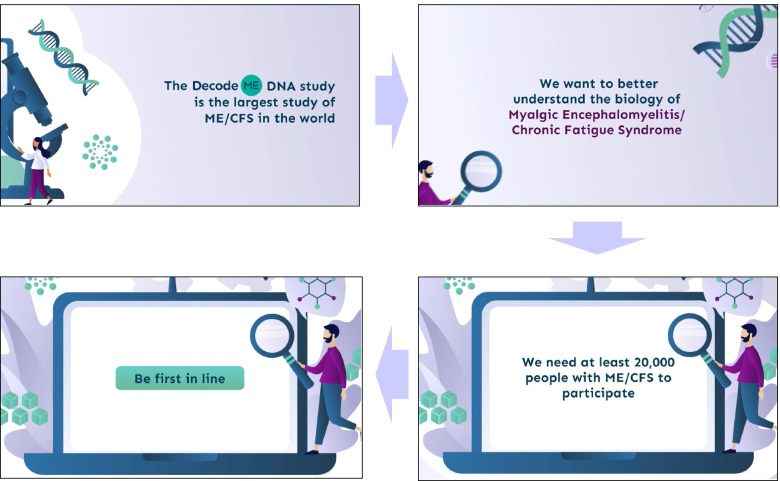
Screenshots from a video released for ME Awareness Week in May 2021: https://www.youtube.com/watch?v=_PIy-1NWHd4. Other videos and webinars are available from the DecodeME YouTube channel

To deliver the cohort size of 25,000 intended participants Itineris, the DecodeME digital marketing company partner, undertook detailed campaign planning using industry benchmarks. Reaching and converting eligible participants are being achieved by: (i) designing and building an effective website (www.decodeme.org.uk), (ii) providing compelling campaign media assets (Fig. 2), (iii) and using conversion rate optimisation techniques that increase form submission rates.

For recruitment we are employing multiple mail outs by charities, person-to-person recruitment, digital marketing, and media events such as Q&A Webinars. DecodeME is involving people with ME as social media ambassadors (to provide feedback and maximise online recruitment) and local ME support groups. Charities contributing to DecodeME are experienced in providing support, including responding to individuals living with severe forms of the disease or experiencing extremely challenging circumstances, during social media interactions.

### Recruitment projections

(1) 20,000 people with ME/CFS not triggered by SARS-CoV-2. Ahead of launch we predicted that approximately half of the 20,000 participant target would be achieved via social media and charities. This prediction assumed that: (i) 1 in 250 adults in the UK have ME/CFS, (ii) two-thirds of these have a clinical diagnosis, and (iii) our sign-up conversion rate is 43% (92% consent, 82% complete questionnaire, 75% satisfy criteria and 76% return the spit kit). Further, we estimated that the UK online ME/CFS patient community is approximately 35,000 people. We additionally assumed that 90% of these have a clinical diagnosis and that we will achieve a 43% sign-up rate, implying that 9,500 of the 20,000-participant target would be achieved via social media and charities. Because of participant attrition (e.g. incomplete questionnaires and unreturned sample kits) we expected to need to persuade 46,500 individuals to sign up in order to reach our final case participant target of 20,000 people with ME/CFS.

(2) 5,000 people with ME/CFS due to SARS-CoV-2 infection. It is estimated that in December 2021 there were 506,000 people in the UK who self-report Long COVID and who first had (or suspected they had) COVID-19 at least one year previously [18]. Approximately 50% (~ 200,000) of these could meet ME/CFS criteria applied in this study although NHS diagnosis is typically slow. Consequently, we expect to quickly recruit 5,000 of this number for this project via social media and our existing connections to Long Covid Support Groups.

### Sample size calculation

Our choice of 25 k study participants was informed by results of similarly-sized GWAS for other conditions and the expected genetic heterogeneity of ME/CFS. Our intended 25 k cohort size provides over 80% power to detect significant (*p* < 5 × 10^–8^) associations for common alleles (population frequency > 5%) increasing risk by 20%, or with lower (~ 1.1) relative risk and reasonably high (> 15%) allele frequency and control-to-case ratio of 4-to-1. GWAS for common conditions that have been performed to date have shown that common allele (allele frequency > 5%) effect sizes often lie in the range of 1.05–1.20 (relative risk, RR), a range for which there is little detection power for studies with fewer than 5,000 cases [19]. Without the benefit of knowing the genetic effect sizes of ME/CFS associations, accurate estimates of the number of loci expected for N = 25,000 cases are not possible. Nevertheless, for a study of 25,000 cases commonly we might expect to find 5 loci and not uncommonly up to 10 loci [20].

### Patient and public involvement (PPI)

DecodeME benefits from real and extensive involvement at every step of the project from people with ME, their carers and ME charities. People with lived experience of ME jointly designed the study, are named Investigators and contribute to each team. The entire project from design-to-delivery is working to PPI National Standards [21]. The benefit from PPI to the project has always been substantial and diverse, and makes for more effective research (Table 1). DecodeME is a co-production involving scientists, people with ME/CFS, carers, charity and industry professionals and members of the public. The study benefits from a Scientific Advisory Board whose members have diverse clinical and non-clinical, ME/CFS and genetics expertise. As required for a co-production [22], DecodeME research is jointly owned and all project members, irrespective of their background, work together to attain common aims. The study would not have been possible without either academic scientists or people with lived experience of ME/CFS. Understanding of the DecodeME project has been enhanced immeasurably by PPI writing public-facing documents and media items. We are actively recording our implementation of co-production and its impact, and will report these elsewhere.

**Table 1:** Key principles of Co-production in the DecodeME study

Principle	Example in DecodeME
Sharing of power	Parity among members of the project’s Management Group: a person with ME/CFS, a charity CEO and a scientist
Including all perspectives and skills	PPI representatives equally contribute to every team meeting and decisions. Managing the pace of the study to ensure that all team members can contribute fully
Respecting and valuing the knowledge of all those working together on the research	Postponement of a submission to a research ethics committee when it became evident that documents written by scientists could be improved substantially by people with ME taking advantage of their lived experience
Reciprocity	Documents and messages open to all project partners
Building and maintaining relationships	People with ME/CFS lay at the heart of DecodeME’s inception, planning and launch

### Preparations for launch

**Fig. 3:**
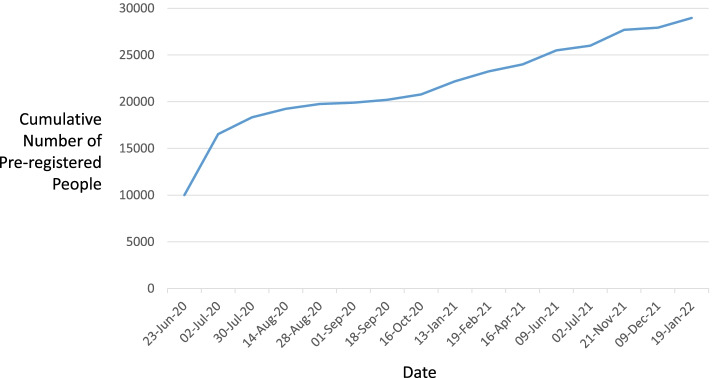
Numbers of registration sign-ups over time. Prior to DecodeME study launch, 28,966 people pre-registered to participate (19 January 2022)

**Fig. 4:**
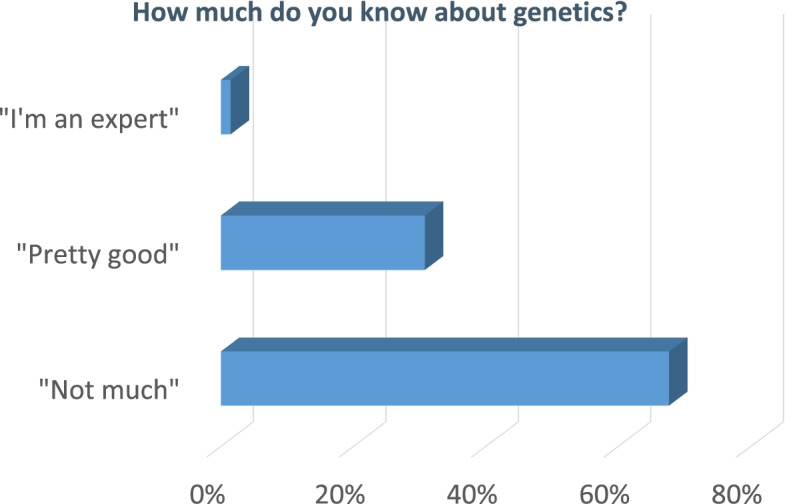
Most respondents know little about genetics. Answers to a Twitter Poll launched by twitter.com/DecodeMEstudy on 18 June 2021. Its question was “How much do you know about genetics?” The poll received 133 votes

Draft documents, including the Participant Information Document, in support of a Research Ethics Committee (REC) submission were rewritten by PPI members to be made more accessible. Favourable opinions from the North West—Liverpool Central REC were received on 14 June 2021 and, in response to a substantial amendment, on 20 October 2021 (Milestones 1 [M1] and M9). DecodeME is registered in the Research Registry (identifying number 6395). Isohelix™ Genefix™ DNA collection kits were procured [M2]), online and paper questionnaires designed [M3, M4]. Data management and security [M5], recruitment planning [M6] and process implementation (including SOPs, strategy documents and communication guidelines) [M7] were all completed prior to PPI-led tests [M8] of the registration, consent and sample collection processes that revealed minor issues which were then rectified. Prior to project launch 29,000 people with ME/CFS in the UK signed up to being contacted (Fig. 3). Public awareness of the project was enhanced by regular podcasts, webinars, blog posts and media interviews. This was needed because two-thirds of respondents in a Twitter poll indicated that they knew little about genetics (Fig. 4). DecodeME also works with the 25% M.E. Group whose members provide phone support to assist people with severe ME/CFS symptoms in filling out their online or paper-based questionnaires.

### Data analysis plan

Analysis and quality control (QC) of Axiom genotype data derived from DNA of ME/CFS cases will be undertaken in large (> 5 × 10^3^) batches as previously described for the UK Biobank data [15]. An initial quality control will consider only variants of minor allele frequency (MAF) ≥ 1% whereas a later analysis, once all 25 k cases have been genotyped, will consider all variants whose minor allele count (MAC) ≥ 20. Genotype calls will be required to be concordant across two standard samples (cell lines of known genotypes) processed repeatedly alongside 94 participant samples, as described previously [15]. Cases and/or controls will be analysed for close-relatedness [23] and genetic ancestry, and 1,000 UK Biobank control image files will be analysed alongside those from cases to minimise spurious associations arising from the separate genotyping of cases and controls.

The main GWAS for 20 k ME/CFS cases will interrogate all good-quality genotyped or imputed common and low frequency variants (MAF ≥ 1%) using imputed data. Summary statistics from this analysis will be made available and used for all downstream analyses (by us and others) including pathway/tissue-type enrichment analyses, and genetic correlations with other traits and diseases [24]. For sensitivity analyses we will test for SNP associations with: (i) ME/CFS case status (NAM/IOM or Canadian Consensus Criteria), and (ii) case severity, based on direct questioning of life impact of disease on participant. Additional tests will be performed for associations with either HLA haplotypes [25] or known copy number variants and rare variants (MAF < 1%, MAC ≥ 20) individually and in burden tests.

For potential internal validation of associations with common alleles (> 30%), detectable with 80% power in a reduced sample, we will undertake the GWAS using 20 k ME/CFS cases in two stages, analysing first only 60% of the cases (~ 12 k cases). Significant (*p* < 5 × 10^–8^) associations will be examined in the subsequently collected 8 k cases, using the lower significance threshold determined by the number of tests (number of loci put forward) performed. GWAS results for the two stages will be combined by meta-analysis into a single, discovery, ME/CFS GWAS. Any associated SNPs discovered will be investigated for independent replication using separately recruited cohorts such as *23andMe* [26] and *Genes for Good* [16], which each include a question on CFS diagnosis. The 5 k post COVID ME/CFS cases would be analysed separately using GWAS and then by comparison of top associations from pre- or post-COVID cases’ GWAS. Finally, a meta-analysis of both pre-and post-COVID ME/CFS will be performed, with shared genetic risks expected to lead to more strongly supported associations.

### Electronic health record (EHR) linkage

DecodeME seeks consent from participants for linkage to their EHR, but this is not a pre-condition for participation in the study. In 2023–24, an EHR pilot project of the expected ~ 10% of DecodeME participants living in Scotland will be undertaken. We will organise and summarise the resulting EHR morbidity data (SMR01) and any other relevant available datasets to inform future EHR linkage analyses across the entire cohort. The frequency of ME/CFS ICD-10 codes in this DecodeME sub-cohort will be analysed and compared with EHR data on matched population controls, such as from Generation Scotland [27] or UK Biobank [15].

## Discussion

### Strengths and limitations

DecodeME is recruiting ten-times more ME/CFS cases than previous genetics studies, resulting in increased statistical power to detect significant genetic differences compared to unaffected controls.

It benefits from community recruitment across the whole of the UK, including people with ME/CFS who rarely engage with the National Health Service. The study is a co-production, being delivered by, for and with people with lived experience of ME/CFS. Use of internationally accepted inclusion criteria for ME/CFS is a strength, but its heterogeneous symptoms mean that not everyone diagnosed with ME/CFS will meet the study criteria. DecodeME will also compare the genetics of ME/CFS triggered by COVID-19 virus infection and ME/CFS due to other causes.

### Future research and collaborations

**Fig. 5:**
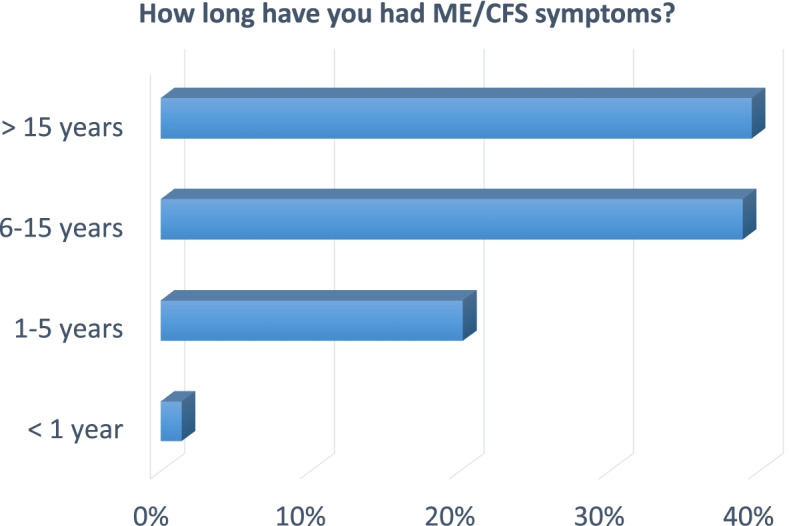
Over 75% of respondents have experienced ME/CFS symptoms for over 6 years. Answers to a Twitter Poll launched by twitter.com/DecodeMEstudy on 23 September 2021. Its question was “If you have ME/CFS, how long have you had #MEcfs symptoms? Or how long as the person you care for…?” The poll received 1,166 votes

Only approximately half of a participant’s DNA sample will be used for genotyping of common DNA variants. The other half will be stored by the NIHR Biosample Centre for future whole genome sequencing (WGS) when funding allows. WGS would provide a complementary approach as it would permit rare DNA variants to be analysed for their association with ME/CFS. DecodeME questionnaire responses should be of substantial epidemiological value, for example regarding symptom duration (Fig. 5). To accelerate ME/CFS research, for participants who have given their informed consent, the DecodeME genotype and phenotype data will be available, via controlled access, to all bona fide researchers whose studies have ethics approval. The DecodeME team welcomes collaboration but this will not be a pre-condition of data access.

## Data Availability

The data that support the findings of this study are available on request from the corresponding authors (ADC, CPP, SC) via a Data Access Committee. The genetic data are not publicly available as this would compromise research participant privacy/consent and some participants have not provided their consent for their data to be shared. At the soonest opportunity, genetic data will be disseminated widely as associated variants and genomic intervals, and as summary statistics via GWAS Catalog (www.ebi.ac.uk/gwas/). Results will be reported on the DecodeME website (www.decodeme.org.uk) and via open access publications. Study documents are also available from this website. People can register to participate in the DecodeME project via the study website or via a paper-based alternative.

## Abbreviations

CMRC: CFS/ME Research Collaborative
EHR: Electronic health records
GWAS: Genome-wide association study
IOM/NAM: Institute of Medicine / National Academy of Medicine
ME/CFS: Myalgic encephalomyelitis / chronic fatigue syndrome
PEM: Post-exertional malaise
PPI: Patient and Public Involvement
WGS: Whole genome sequencing
